# Fundamental Study of a Wristwatch Sweat Lactic Acid Monitor

**DOI:** 10.3390/bios14040187

**Published:** 2024-04-10

**Authors:** Sakae Konno, Hiroyuki Kudo

**Affiliations:** Department of Electronics and Bioinformatics, School of Science and Technology, Meiji University, Tokyo 214-8571, Kanagawa, Japan

**Keywords:** wearable, sweat, electrochemical, microfluidic, biosensor

## Abstract

A lactic acid (LA) monitoring system aimed at sweat monitoring was fabricated and tested. The sweat LA monitoring system uses a continuous flow of phosphate buffer saline, instead of chambers or cells, for collecting and storing sweat fluid excreted at the skin surface. To facilitate the use of the sweat LA monitoring system by subjects when exercising, the fluid control system, including the sweat sampling device, was designed to be unaffected by body movements or muscle deformation. An advantage of our system is that the skin surface condition is constantly refreshed by continuous flow. A real sample test was carried out during stationary bike exercise, which showed that LA secretion increased by approximately 10 μg/cm^2^/min compared to the baseline levels before exercise. The LA levels recovered to baseline levels after exercise due to the effect of continuous flow. This indicates that the wristwatch sweat LA monitor has the potential to enable a detailed understanding of the LA distribution at the skin surface.

## 1. Introduction

Biochemical information has become increasingly important for the Internet of Health Things (IoHT), especially after the COVID-19 pandemic [1,2,3]. Eccrine sweat, which contains a variety of biomarkers, has received considerable attention as a target for noninvasive, continuous, and simplified biomonitoring. Measuring sweat components is useful for disease diagnosis, healthcare, and exercise management, and it is expected to play a crucial role in various fields, including point-of-care testing [4,5]. Chloride ions in sweat are measured for diagnostic purposes, such as in newborn screening to diagnose cystic fibrosis [6,7]. In contrast, for exercise management purposes, electrolytes such as sweat sodium are considered crucial markers for maintaining and improving performance and preventing dehydration [8,9]. Other components of sweat, such as lactic acid (LA) and glucose, have also been studied for their potential applications in exercise management and healthcare [10].

Despite potential applications, the accurate measurement of sweat components remains a challenge for several reasons, including the highly variable sweat rate, complex secretion mechanisms involving reabsorption, evaporation after secretion from sweat glands, and interactions between components and bacteria (which can reach a density of approximately 10 billion/cm^2^ on the skin surface [11] immediately after sweating). For example, LA secretion from sweat glands is mainly determined by the metabolic activity of sweat gland myoepithelial cells and is dependent on the amount of sweat. However, it cannot be measured based on the sweat rate, and its kinetics remain unclear [12,13]. The simultaneous real-time monitoring of sweat LA, sweat rates, and other biological information under various conditions can provide a better understanding of the factors that influence lactate secretion into sweat. This approach allows for a detailed study of the correlation between these factors. The most common approach for measuring sweat components is to analyze them after collecting sweat for a fixed time. Recently, highly complete wearable devices have been developed that can measure sweat components directly at the skin surface [14,15]. In addition, there are reports of many sophisticated devices capable of multi-sensing sweat components, resulting in much more convenient and accurate real-time sweat monitoring [16,17,18,19,20,21,22]. Although sweat monitoring systems have advanced, the aforementioned problems have not yet been completely solved. In particular, it is still difficult to measure sweat components under low perspiration because the device, which uses capillary action or sweating pressure, requires high perspiration to collect and transport sweat to the sensor. This limits measurement opportunities to situations where it is relatively easy to increase perspiration, such as during bike exercise [23,24,25,26]. The measurement conditions have been greatly expanded by the development of devices that actively collect sweat and measure its composition using iontophoresis, which induces perspiration by switching the activity of sweat glands by electrically inducing pilocarpine ions [27,28,29]. From the point of view of daily personal use for healthcare, further breakthroughs are still required to enable sweat monitoring under large sweat rate fluctuations.

We developed a wristwatch-type biosensing system that can continuously measure LA secreted from the skin surface in real time, independent of the sweat rate [30,31], and its fundamental characteristics are investigated in detail. In this system, phosphate-buffered saline (PBS) is perfused over the skin at a constant rate as carrier flow to actively transport secretions from the skin surface to the downstream biosensor. The advantage of our system is that the influence of the sweat rate is minimized, and the initial state of the skin surface is maintained throughout the measurement process. This paper describes the updates from a previous study [31], including the detailed structure and characteristics of the wristwatch sweat LA monitoring system, which has an enzyme biosensor and a fluid control mechanism for taking measurements in situations involving the body movement of the subject.

## 2. Materials and Methods

### 2.1. Reagents

L-lactic acid (CAS: 79-3344, 129-02666), sodium chloride (CAS: 7647-14-5, 195-15975), potassium dihydrogen phosphate (CAS: 7778-77-0,164-22635), disodium hydrogen phosphate (CAS: 7558-79-4, 042-30055), urea (CAS: 57-13-6, 215-00172), ethanol (CAS: 64-17-5, 057-00456), D-glucose (CAS: 50-99-7, 049-31165), L-valine (CAS: 72-18-4, 228-00082), L-leucine (CAS: 61-90-5, 124-00852), and ammonia (CAS: 1336-21-6, 013-17505) were all purchased from Wako, Japan. Lactate oxidase (LOD) from *Aerococcus viridans* (T-47, Asahi Kasei Pharma, Tokyo, Japan) was purchased from Asahi Kasei Pharma and was used to modify the electrode of the biosensor. Osmium-wired horseradish peroxidase (002096, Bioanalytical Systems, West Lafayette, IN, USA) was purchased from BAS Inc., Tokyo, Japan. Phosphate-buffered saline (PBS, pH 7.4, 50 mM (PO_4_)) was prepared in the laboratory. Ultrapure water obtained from the water purification system (Direct-Q, Merck, Darmstadt, Germany) was used for the preparation of all aqueous solutions.

### 2.2. Construction of the Sweat LA Monitor

**Figure 1:**
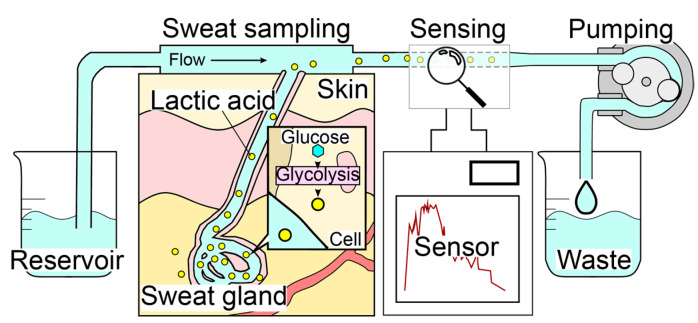
Mechanism of the real-time sweat monitoring system. Carrier flow is perfused over the skin to collect secretions, which are continuously measured by the LA biosensor. The skin surface condition is constantly refreshed by the continuous flow.

**Figure 2:**
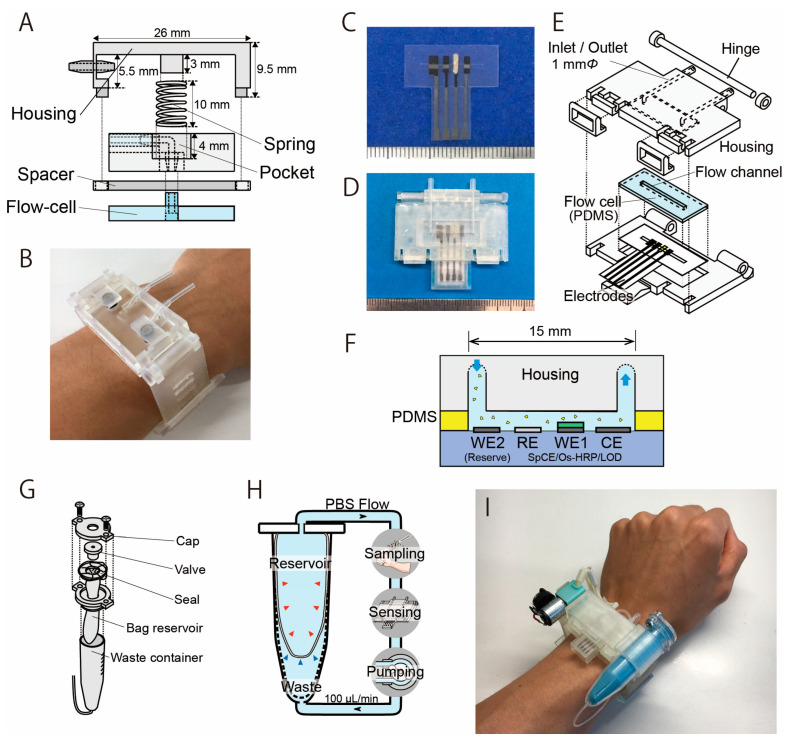
Structure of the sweat sampling unit and the LA biosensor with a microflow channel. (**A**) The sweat sampling unit has a spring that is used to press the PDMS flow cell with the skin. (**B**) Photograph of the sampling unit attached on the wrist (modified from Ref. [31]). (**C**) Photograph of the LA biosensor. From left: counter electrode, working electrode, Ag/AgCl reference electrode, and reserve electrode. The electrodes were covered with an insulator, except for the 1 mm wide sensing region. (**D**) Biosensing unit containing the PDMS flow cell and the electrodes. (**E**) Structure of the biosensing unit. (**F**) Cross-section image of the sweat LA biosensor. (**G**) Structure of the reservoir unit. (**H**) Mechanism of the dual-cell reservoir. The reservoir cell shrinks as the PBS decreases, and the waste cell expands accordingly. (**I**) Photograph of the wearable sweat LA monitor.

The sweat LA monitor consists of three elements: a sweat sampling unit, an electrochemical LA biosensor, and a carrier flow controller (Figure 1). The system employs a carrier flow on the skin surface, which is supplied through a PDMS flow cell. Sweat secreted on the skin surface is immediately dissolved in the continuous carrier flow of PBS and transported to the LA biosensor that is highly specific to LA. The use of carrier flow minimizes the influence of varying chemical conditions caused by evaporation, accumulation, and bacterial activity at the skin surface. The pump downstream of both the sweat sampling device and the LA biosensor regulates the flow rate, and the total flow rate of the carrier flow containing sweat remains constant (i.e., the flow rate of the carrier flow depends on the sweat rate). Therefore, our method was found to be highly accurate in monitoring the total LA secretion (μg/cm^2^/min) rather than the LA concentration in sweat, which can be estimated by dividing the total secretion rate by the sweat rate. In order to perform continuous LA monitoring during exercise, the wristwatch-type sweat LA monitor was designed to allow movement in the subject’s body (Figure 2).

#### 2.2.1. Sweat Sampling Unit

Figure 2A shows the structure of the sweat sampling unit, which consists of a PDMS microflow cell and housing unit that allows the flow channel to be attached to the skin surface. The central spring shown in Figure 2A applies appropriate pressure to the PDMS microflow cell against the skin surface. The flow channel is 15 mm × 2 mm (depth: 1 mm) and was molded on a 4 mm thick PDMS using a conventional molding process. The housing unit was fabricated using a stereolithography rapid prototyping system with urethane dimethacrylate (UDMA) resin (Figure 2B). Flexible elastic UDMA resin (FLELCL01, Formlabs, Boston, MA, USA) was employed as the structural material for the watch band.

#### 2.2.2. Electrochemical LA Biosensor

The design and appearance of the LA biosensor are shown in Figure 2C–E. It was prepared similarly as explained in our previous literature [30,31]. The biosensor measures LA as a product of hydrogen peroxide resulting from the LOD reaction that catalyzes the oxidation of LA. The production of hydrogen peroxide was detected using a three-electrode method based on the redox system of an osmium-wired horseradish peroxidase (HRP). The LA biosensor has a carbon–graphite (C2130809, Gwent Electronic Materials, Derbyshire, UK) working electrode (WE1) modified with LOD (immobilized in polyvinyl alcohol), a counter electrode (CE), and a silver/silver chloride reference electrode (RE) (Figure 2C). All electrodes were prepared using the conventional screen-printing method. The device uses a silver/silver chloride reference electrode that is formed on a carbon electrode. Unlike a conventional reference electrode, this electrode is not separated by a porous filter; we confirmed that this configuration does not significantly impact the measurements, and this is also discussed in Section 3.1. The LOD enzyme was immobilized on the electrode by encapsulating it in a polymer matrix of hydrophilic photo-cross-linkable polyvinylalcohol. On the working electrode, 0.2 μL of osmium-wired horseradish peroxidase (002096, Bioanalytical Systems, West Lafayette, IN, USA) was applied. Subsequently, 0.2 μL of an enzyme solution was prepared using 1 mg of LOD (T-47, Asahi Kasei Pharma Co., Tokyo, Japan), 10 μL of PBS, and 60 μL of UV-curing resin (Biosurfine-AWP-MRH, Toyo Gosei Co., Tokyo, Japan). The solution (0.2 μL) was then applied to the sensing region and incubated at 4 °C for 10 min. The LA biosensor was constructed by fixing the electrode and a polydimethylsiloxane (PDMS) (SYLGARD^®^ 184 SILICONE ELASTOMER KIT, Dow Corning, Midland, MI, USA) microflow cell in a sensor housing unit with a microflow channel (Figure 2D). In order to maximize the reproducibility and simplify the mounting procedure, the sensor housing has a hinge structure for attaching the microfluidic channel to the LA biosensor. This structure automatically aligns the microfluidic channel to the sensing region of the LA biosensor (Figure 2E). The housing was fabricated using a stereographic rapid prototyping system (Form3, Formlabs, Boston, MA, USA). UDMA resin (FLGPCL04, Formlabs, Boston, MA, USA) was selected as the housing material. The LA biosensor measures the LA contained in the sample flow in the PDMS flow cell (flow channel: 15 mm × 1 mm) (Figure 2F).

#### 2.2.3. Carrier Flow Reservoir

Figure 2G shows the structure of the carrier flow reservoir. The carrier flow reservoir comprises a flexible dual-cell structure in a waste container (VIO-5B, AS ONE Corporation, Japan). Each cell is separated by a flexible latex film (No882-SS, Showa Glove, Japan) (Figure 2H). Both the connection to the measurement system and the injection of PBS into the reservoir occur via a valve (BC0043-W, Molten, Tokyo, Japan). The carrier flow supplied from the reservoir cell is collected in the waste cell after passing through the sweat sampling device and the LA biosensor throughout the measurement process. The latex film of the dual-cell structure deforms flexibly to reduce the fluctuation in internal pressure.

#### 2.2.4. Wristwatch Sweat LA Biosensor

The components (biosensor, sweat sampling device, reservoir, and other elements) were assembled into a wristwatch-type housing unit (Figure 2I and Figure S2). The flow channel of each component was connected using a silicone tube (with an inner diameter of 0.5 mm). The LA biosensor was placed directly above the sweat sampling unit to minimize the delay due to sweat transport from the sampling unit to the LA biosensor. The channel length from the sampling unit to the LA biosensor was 30 mm, and the carrier flow was perfused at a rate of 100 µL/min. Therefore, the time required to transport the secreted sweat to the LA biosensor was approximately 15 s. With the help of the 3D-printed housing, it took less than 20 s to become ready for the measurement stage by changing the disposable electrodes.

### 2.3. Characterization of the Wristwatch Sweat LA Biosensor

We first investigated the electrochemical properties, including the responses to LA and specific activity, of the LA biosensor in vitro. Currents corresponding to the products of enzymatic reactions were recorded using an electrochemical analyzer (ALS701Es, CH Instruments, Austin, TX, USA). During the measurement stage, test solutions were delivered at a flow rate of 100 μL/min using a micropump (RP-Q1.2C-P20Z-DC3V, Aquatech, Osaka, Japan). The biosensor has a screen-printed carbon electrode and a silver/silver chloride reference electrode in the same flow channel without any liquid junction. We confirmed that the expected changes in NaCl levels, due to perspiration, do not result in any serious effect on sensor stability or output current. We also confirmed that changes in the angle of the reservoir, which can occur with movement of the subject, do not have a significant effect on the internal pressure of the flow channel and tubing (Figure S1). Finally, we investigated how the output of the sensor worn on the subject’s wrist was affected by wrist and arm movement. In this test, a thin PDMS membrane was placed between the skin and the sampling unit to eliminate the effects of sweating or other changes in skin conditions.

### 2.4. Real Sample Test

Real-time LA monitoring was performed in healthy adult male subjects under the approval of the Ethical Committee of Meiji University. The forearm was carefully rinsed with milli-Q water to remove skin debris prior to wearing the device. Subjects rested sufficiently before and after completing the exercise. In the experiment, subjects wore a complete set of the wristwatch sweat LA monitor and then turned on the micropump to perfuse the carrier flow. A wireless potentiostat (μstat400, Metrohm DropSens, Oviedo, Spain) used for measurement purposes was worn on the back in a waist bag, and the output current was sent to a laptop PC via Bluetooth. During LA measurement, a micro-perspiration meter (TPL3520, TECHNO NEXT, Chiba, Japan) was attached to the subject’s forearm near the sweat sampling site to simultaneously measure sweat volume while monitoring LA levels.

## 3. Results and Discussion

### 3.1. Characteristics of the LA Biosensor

**Figure 3:**
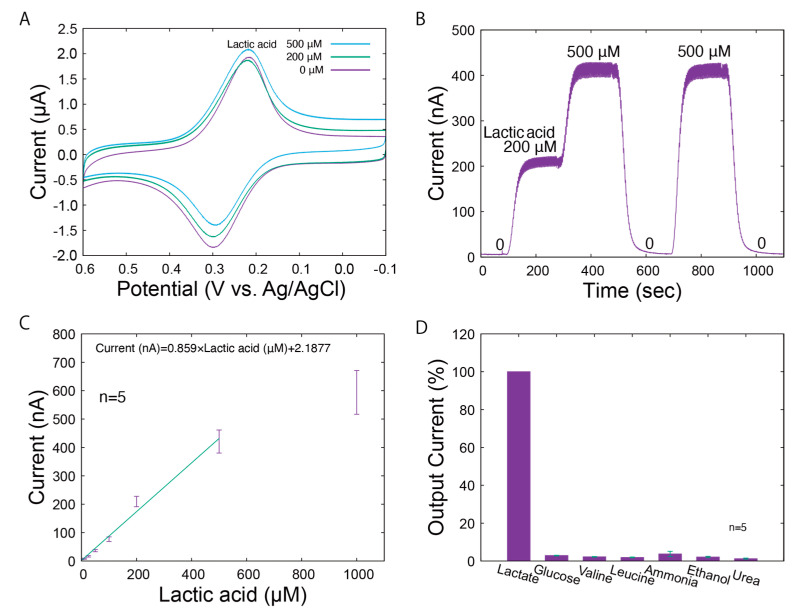
Characteristics of the LA biosensor. (**A**) Cyclic voltammograms for LA (0, 200, and 500 μM LA) dissolved in PBS. (**B**) Amperometric responses to various concentrations of LA. (**C**) The calibration curve for standard LA solutions. The error bars represent the standard deviations. (**D**) Selectivity of the LA biosensor.

The LA biosensor was characterized using conventional amperometric techniques. The electrochemical properties of the LA biosensor are shown in Figure 3. As observed in the cyclic voltammograms under different LA levels (Figure 3A), the reduction currents peaked around 0.2 V vs. Ag/AgCl. The reduction currents indicate that there is electron transfer between the mediator at WE1. Diffusion-independent reduction currents were identified around 0 V vs. Ag/AgCl, which reflect the concentrations of LA. Therefore, we operated the LA biosensor at 0 V vs. Ag/AgCl. Typical responses of WE1 to the changes in LA levels (0, 200, and 500 μM) are shown in Figure 3B. The output current immediately increased according to the LA concentrations and returned to the initial value in the absence of LA. To demonstrate the behavior of our system under real conditions, the responses shown in the figures include periodic noise generated by the pulsation of the micropump. This noise can be eliminated by applying a low-pass filter. In addition, for the LA biosensor, a constant baseline of output current was maintained for each LA concentration. The current levels under the different LA concentrations are shown in Figure 3C. The linear range was 25–500 µM, and the sensitivity was 0.859 nA/µM. Based on the sweat on the forearm surface (sweat rate: 1.5 mg/cm^2^/min; sweat LA: 5 to 60 mM [5]) and the carrier flow rate (100 μL/min, window: 0.3 cm^2^), the sweat was diluted approximately 200 times in the carrier flow during the measurement stage. This indicates that the calibration range is sufficient for sweat LA monitoring.

The selectivity of the LA biosensor was also evaluated. LA and chemicals expected to be present in the sample (glucose, valine, leucine, ammonia, ethanol, and urea) were prepared at 0.5 mM each, and the output current was recorded when introduced into the LA biosensor. Figure 3D shows the relative output currents for each solution compared to that of LA. The biosensor demonstrates high specificity for LA, which can be attributed to the specific activity of LOD from *Aerococcus viridans*. We also evaluated the influence of possible changes in ion concentration on sweat monitoring, in addition to selectivity based on the specific activity of LOD. The effect of sodium chloride concentrations on the biosensor was studied, which confirmed that possible changes in sodium chloride levels, both increases and decreases, do not significantly affect the current output (Figures S3 and S4). However, it is preferable that the skin surface is sufficiently washed in advance. The impacts of sodium chloride levels under more practical conditions (carrier flow: 100 μL/min; window: 0.3 cm^2^; sweat rate: <3.0 mg/cm^2^/min; and sodium chloride: 10~100 mM [5]) were almost negligible. In this system, sweat is transported by a carrier flow that is approximately 100 to 200 times higher than the sweat rate. The concentration of chemicals in PBS, such as chloride ion, remain relatively stable despite changes in the sweat rate and contents. However, the biosensor can directly measure the total secretion of lactic acid, which is not contained in the carrier flow. From the above results, we conclude that our biosensor has characteristics suitable for monitoring LA in sweat.

### 3.2. Impacts of Body Movements

**Figure 4:**
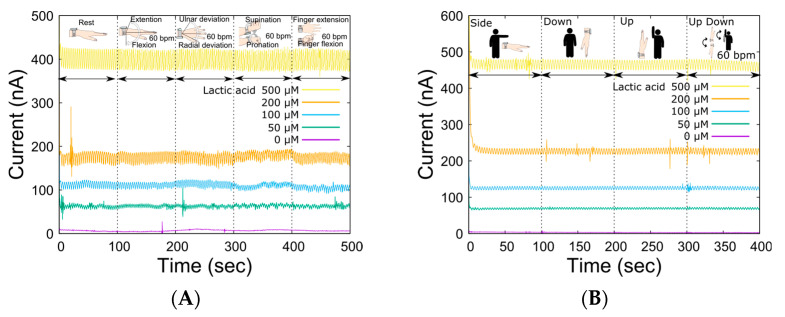
Fluctuations in the sensor output due to various actions and movements of the body. (**A**) Changes in sensor output for various LA levels in response to joint movement (wrist: extension and flexion, ulnar and radial deviation, supination, and pronation; finger: extension and flexion, 60 BPM). (**B**) Impact of device angles resulting from arm movement.

In LA monitoring using our system, the open-channel flow cell was placed on the skin, and sweat was collected by perfusing the carrier flow into the channel. Therefore, the compression force of the sampling device was controlled by an elastic spring force to allow sweat sampling to be performed regardless of complex skin surface deformation due to movements around the wrist joint. The spring constant was optimal at 0.476 N/mm, considering the trade-off between reduced carrier flow leakage and flexibility against changes in skin surface conditions caused by skeletal movements associated with each movement [31]. Table 1 shows the specifications of the wristwatch LA monitor. The device could be worn by the subject alone, and the total weight of the device was 67 g (except for carrier flow), which is sufficiently light for exercising. Then, we investigated the output change in the LA biosensor caused by movements around the wrist joint (Figure 4A) to ensure sweat LA monitoring during the bike exercise. There was no significant change in the baseline output current for each LA concentration due to movement around the wrist joint. Although the effects were almost negligible for each movement, the output fluctuated the most during supination and pronation. This was due to the large movement of the radius and ulna during wrist rotation, which resulted in large deformations in the skin surface. The dependence of the output current on the system angle was also measured (Figure 4B). As shown in the figure, there was less variation in the output current baseline than in the wrist movement. These results suggest that the liquid line based on the optimized reservoir and the microtube pump contributes to the stabilization of sample transport. Thus, the ability of the wristwatch device to monitor LA secretion independent of the subject’s movement was confirmed. In microsystem research, it is common to consider device miniaturization as a strategy to reduce undesired effects, such as from wrist movement. However, we decided to use such a system because the influence of the few covered sweat glands would be much more significant.

### 3.3. Sweat LA Monitoring

The source of skin LA is the LA produced by bacterial activities and LA that accumulates in the stratum corneum [32]. In contrast, sweat gland myoepithelial cells are the primary source of LA in sweat. Our system samples both types of LA simultaneously from the skin surface. Hence, it is important to consider the impact of skin LA in sweat LA monitoring. To isolate the signal corresponding to skin LA, washing the skin is effective. Since our system perfuses PBS on the skin surface, the skin is constantly being washed during the measurement stage. If skin cleansing is not performed, the output typically decreases sufficiently after 15 to 20 min of PBS perfusion at the skin surface. During the cleaning phase, skin LA can be monitored even without sweating (Figure S5). Skin LA found at the beginning of the monitoring is drastically reduced after this (Figure S6). The amount of LA secretion on the skin is then calculated from the calibration curve and sampling area on the skin using the carrier flow rate. The sum of carrier flow and sweat rate is always constant (100 μL/min). Since the carrier flow does not contain LA, all LA detected by the biosensor is recognized after being secreted from the skin. The amount of LA (μg) passing through the flow channel per minute is calculated from the LA level measured by the biosensor and the pumping rate, and then divided by the window size of the sampling device to obtain the amount of total LA secretion (μg/cm^2^/min).

**Figure 5:**
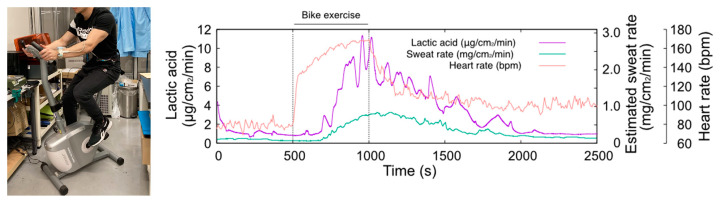
Photograph of the sweat LA monitoring during bike exercise (**left hand**) and the changes in heart rate, sweat LA, and estimated sweat rate (**right hand**). The temporal change in sweat LA follows the trend of the sweat rate.

Figure 5 shows the results of LA monitoring, sweat rate, and heart rate during the stationary bike exercise. The maximum heart rate of this exercise task was approximately 160 BPM and lasted for 500 s, which is a sustainable exercise intensity. The sweat rate significantly increased around 200 s after the start of the exercise and peaked at the end of the exercise. The TPL3520 sweat rate monitor was influenced by humidity, which was compensated for by linear fitting. During exercise, LA secretion increased rapidly from the baseline level of 1 μg/cm^2^/min, in line with an increase in the sweat rate. The maximum observed LA level was 12 μg/cm^2^/min toward the end of the exercise. Following the end of the exercise, LA output gradually decreased with a decrease in the sweat rate. After 2000 s, the output returned to the baseline level of about 1 μg/cm^2^/min, the same as before the start of the exercise. The amount of LA secretion on the skin was found to be strongly related to the sweat rate. This experiment confirms that LA secretion increases as the sweat rate ascends. Our system measures the amount of LA dissolved in the carrier flow directly, regardless of the sweat rate or skin condition. Therefore, no individual calibration is required to monitor lactate secretion on the skin surface. From these findings, we concluded that a wristwatch sweat LA monitor can be used to monitor LA secretion on the skin during exercise.

## 4. Conclusions

The development of a sweat LA monitoring system using an electrochemical biosensor based on the redox reaction of lactate oxidase is described. The electrochemical biosensor was embedded on a miniaturized fluid control system, enabling the stable and continuous monitoring of sweat LA during bike exercise. The LA biosensor was fabricated using conventional screen-printing and enzyme immobilization techniques. This indicates that the sweat LA monitor is also useful for monitoring other substances, such as glucose and ethanol, by immobilizing redox enzymes instead of LOD on the working electrode. The mechanisms for fluid control, such as the dual-cell reservoir and spring for flow cell attachment, minimized impacts caused by angular changes in the attachment site. The results of LA monitoring suggest that the kinetics of LA secretion can change significantly depending on the content of exercise. However, the further investigation of LA kinetics in response to exercise is needed. It is expected that the wristwatch-type LA monitoring system can be used in sweat LA monitoring under various conditions to improve the understanding of the LA distribution at the skin surface.

## Figures and Tables

**Table 1:** Specifications of the sweat LA monitor.

Weight	Biosensor: 5.8 g
	Sampling device: 26.8 g
	Reservoir: 5.6 g
	Pump and battery: 14.9 g
	Other components: 13.9 g
	Total: 67 g
Dimension	Biosensor unit: 40 × 35 × 5 mm^3^
	Sampling device: 46 × 26 × 9.5 mm^3^
	Reservoir: 20 × 60 × 25 mm^3^
Setup time	<60 s
Lower detection limit	0.75 μg/cm^2^/min
Measurement time ^1^	50 min
	(@ 5 mL reservoir, 100 μL/min carrier flow)

^1^ Depends on the reservoir volume and the flow rate.

## Data Availability

Data are contained within the article.

## Supplementary Materials

The following supporting information can be downloaded at https://www.mdpi.com/article/10.3390/bios14040187/s1, Figure S1: Inner pressure change against the reservoir angle; Figure S2: Structure of the sweat LA monitor; Figure S3: Current output before and after the increase in NaCl levels; Figure S4: Effect of the decrease in NaCl levels; Figure S5: Typical skin LA signal from the subject without a skin washing procedure; Figure S6: LA monitoring results for a 500 s period.
