# Supplementary material for: Fundamental Study of a Wristwatch Sweat Lactic Acid Monitor
Source: Biosensors (Basel). 2024 Apr 10;14(4):187. doi: 10.3390/bios14040187 (PMC11048019; doi:10.3390/bios14040187)
Supplement: Supplementary file 1 [file biosensors-14-00187-s001.zip › biosensors-2921925-supplementary.pdf]

biosensors

Supplementary materials

Fundamental Study of a Wristwatch Sweat Lactic Acid Monitor

Sakae Konno and Hiroyuki Kudo *

Department of Electronics and Bioinformatics, School of Science and Technology, Meiji University, Kanagawa 214-8571, Japan
* Correspondence: hkudo@meiji.ac.jp

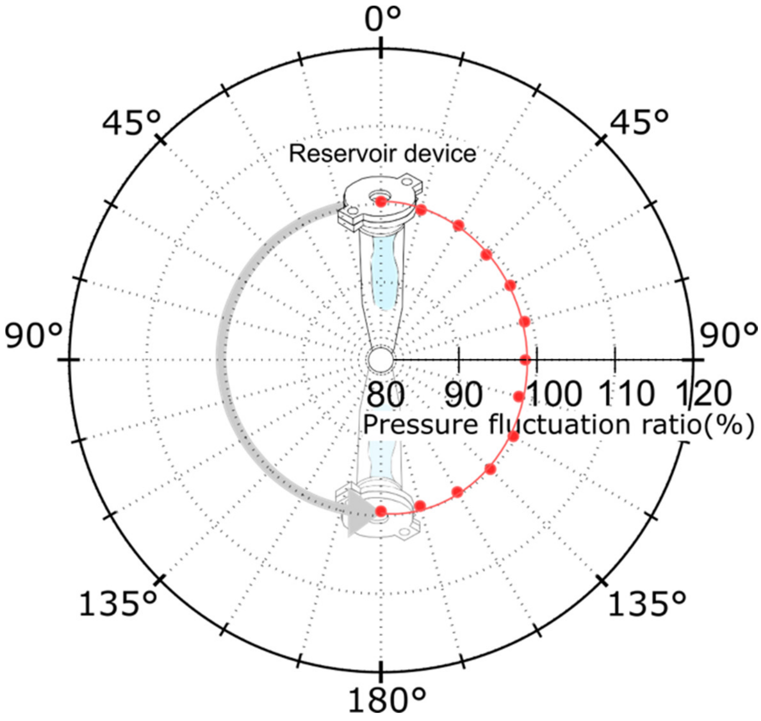

Figure S1. Pressure change against the reservoir angle. Red plot represents the ratio of inner pressure at the outlet of reservoir and the pressure at the inlet of the waste. The pressure ratio is close to 100% at all angles, and this indicates that the effect of reservoir angle on the internal pressure is almost negligible.

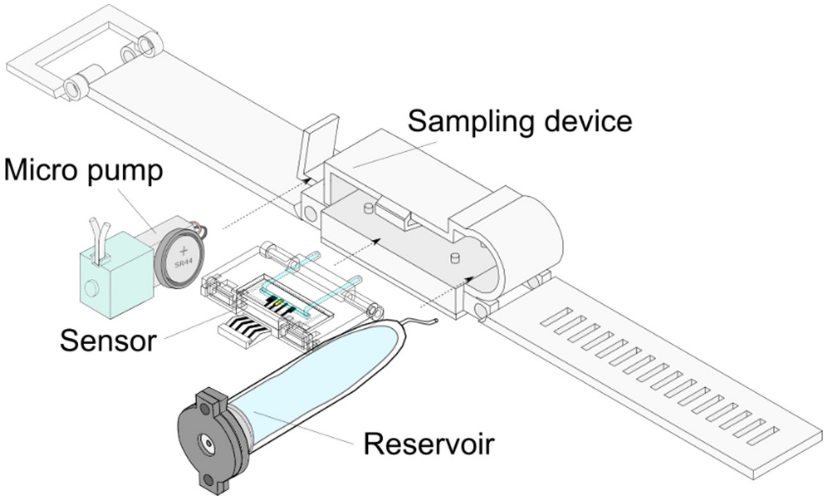

Figure S2. Structure of sweat LA monitor.

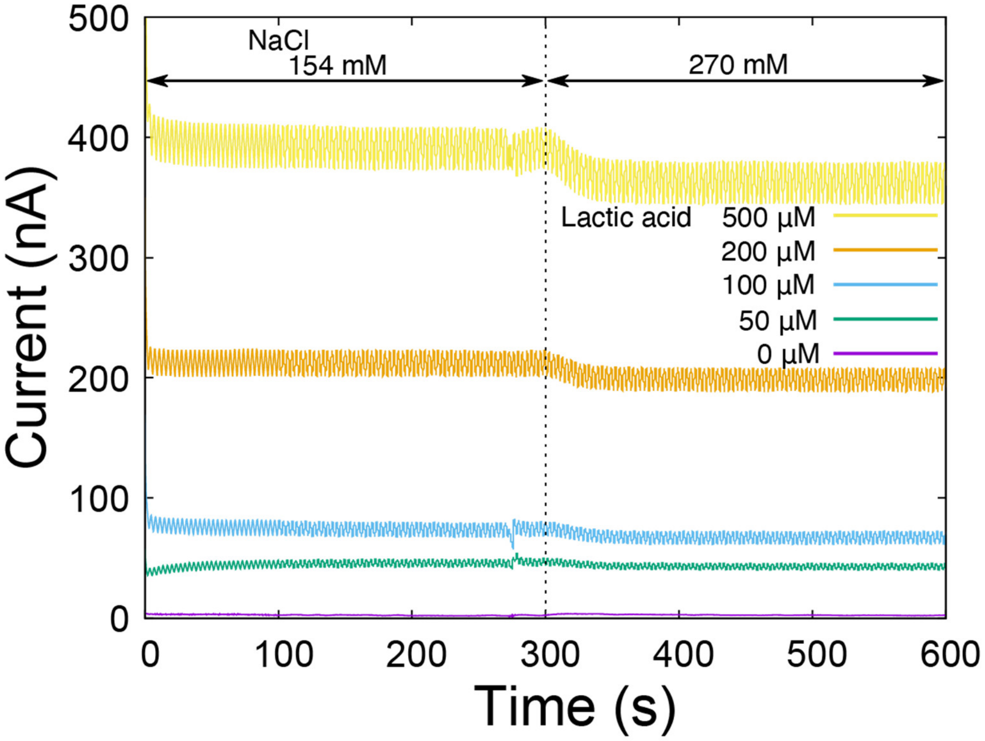

Figure S3. Current output before and after the increase of NaCl level (from 154 mM to 270 mM). Suppose that sweating at an extremely high rate (3.0 mg/cm²/min, NaCl: 100 mM) continues for one hour and sweat sodium chloride all accumulates on the skin due to sweat evaporation, although it is not realistic condition. In this case, the maximum concentration of sodium chloride in carrier flow (flow rate: 100 μL/min, window: 0.3 cm²) due to the accumulation collection is at most 270 mM.

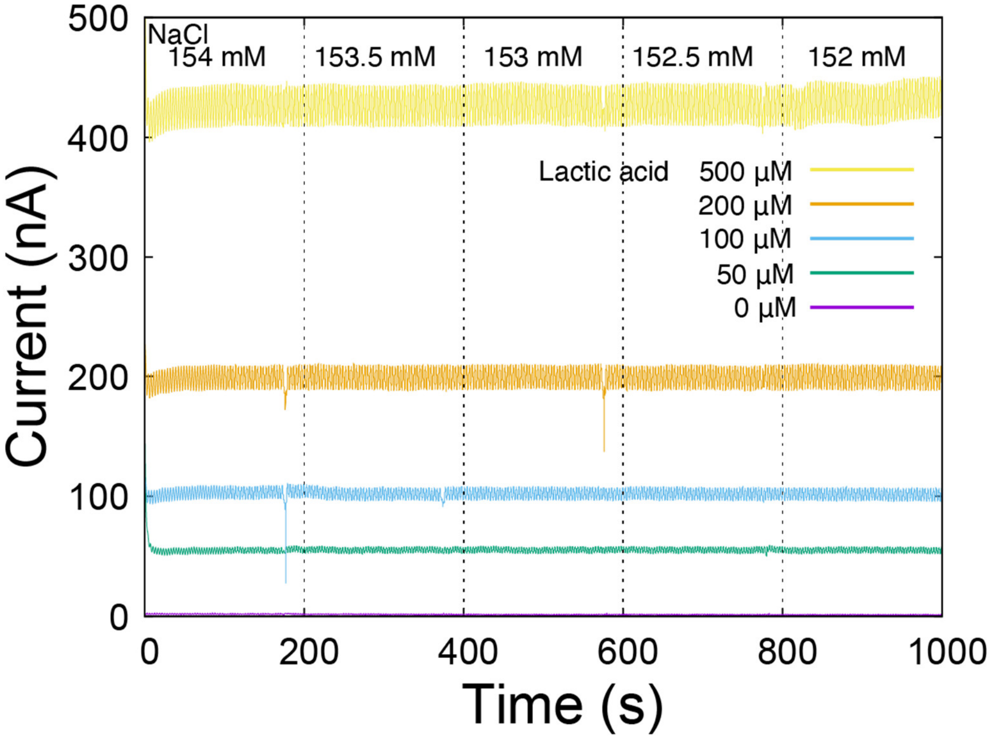

Figure S4. Effect of decrease in NaCl levels. The concentrations were determined from the expected variation of NaCl in the carrier flow due to perspiration.

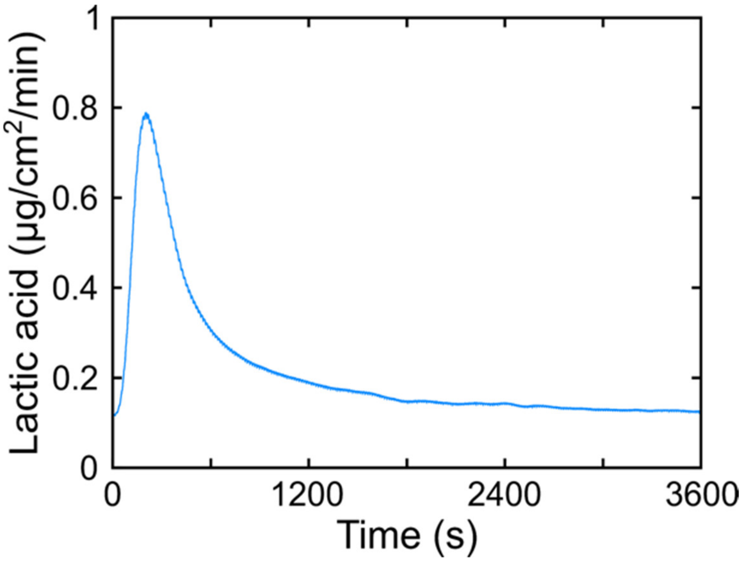

Figure S5. Typical skin LA signal of the subject without skin wash procedure.

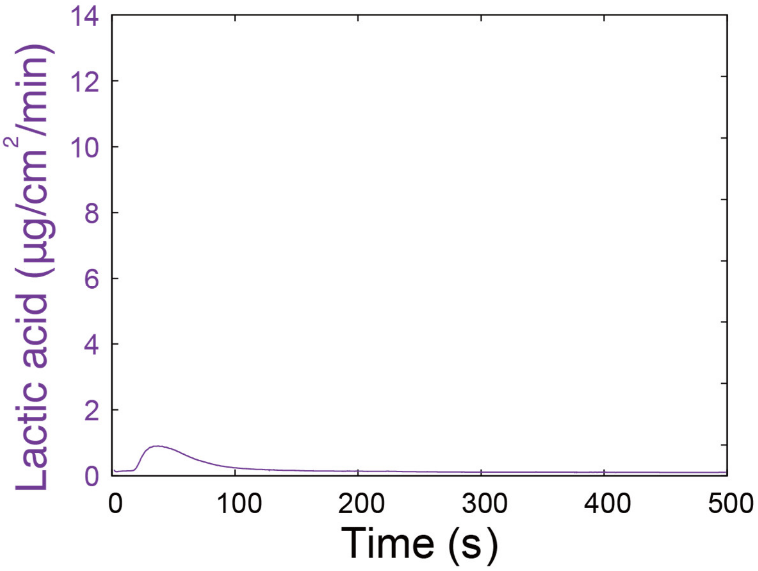

Figure S6. LA monitoring results for a 500-second period while the subject, washed the skin before measurement, was seated and at rest.
